# Network pharmacology, molecular docking, and experimental verification reveal the mechanism of San-Huang decoction in treating acute kidney injury

**DOI:** 10.3389/fphar.2023.1060464

**Published:** 2023-02-06

**Authors:** Jiahui Liu, Zhongtang Li, Yunlan Lao, Xiaoming Jin, Yuzhi Wang, Beibei Jiang, Riming He, Shudong Yang

**Affiliations:** ^1^ Shenzhen Traditional Chinese Medicine Hospital Affiliated to Nanjing University of Chinese Medicine, Shenzhen, China; ^2^ Department of Nephrology, Shenzhen Traditional Chinese Medicine Hospital, The Fourth Clinical Medical College of Guangzhou University of Chinese Medicine, Shenzhen, China

**Keywords:** acute kidney injury, cisplatin, San-Huang decoction, network pharmacology, molecular docking

## Abstract

**Background:** Cisplatin is an effective anti-tumor drug. However, its usage is constrained by side effects such as nephron toxicity. Cisplatin-induced acute kidney injury (AKI) appears in approximately 20%–30% of cases. Hence, finding an effective protective strategy is necessary. San-Huang decoction (SHD) is a Chinese herbal decoction with good efficacy in treating chronic kidney disease (CKD). Nevertheless, the mechanism of SHD on AKI remains unclear. Consequently, we proposed to explore the potential mechanism of SHD against cisplatin-induced AKI.

**Methods:** Active compounds, core target proteins, and associated signaling pathways of SHD were predicted through network pharmacology. Then confirmed by molecular docking. In vivo experiment, Cisplatin + SHD group was treated with SHD (6.5 g/kg/day) for 6 days before building the model. An AKI model was established with a single intraperitoneal injection of cisplatin at 20 mg/kg. After 72 h of cisplatin injection, all mice were sacrificed to collect blood and kidney tissues for verification of network pharmacology analysis.

**Results:** We found that calycosin, rhein, and ginsenoside Rh2 may be SHD’s primary active compounds in treating cisplatin-induced AKI, and AKT, TNF-α, IL-6, IL-1β, caspase-3, and MMP9 are the core target proteins. The relationship between the compound and target protein was further confirmed by molecular docking. The Gene Ontology (GO) and the Kyoto Encyclopedia of Genes and Genomes (KEGG) pathway analyses predicted that SHD has an anti-inflammatory role through the TNF and IL-17 signaling pathway. Moreover, Western blot and immunohistochemistry validated the potential molecular mechanisms of SHD, predicted from network pharmacology analysis. The mechanism of cisplatin-induced AKI involves apoptosis and inflammation. In apoptosis, Caspase-3, caspase-8, caspase-9, and Bax proteins were down-regulated, while Bcl-2 was up-regulated by SHD. The differential expression of MMP protein is involved in the pathological process of AKI. MMP9 protects from glomerular tubule damage. MMP9 and PI3K/AKT anti-apoptosis pathway were up-regulated by SHD. In addition, we discovered that SHD alleviated AKI by inhibiting the NF-κB signaling pathway.

**Conclusion:** SHD plays a critical role in anti-inflammation and anti-apoptosis *via* inhibiting the NF-κB signaling pathway and activating PI3K/AKT anti-apoptosis pathway, indicating that SHD is a candidate herbal drug for further investigation in treating cisplatin-induced AKI.

## Introduction

Acute kidney injury (AKI) is a multifunctional kidney disease characterized by a sudden kidney failure episode, increased serum creatinine levels, and a marked decrease in urine volume, with a significant risk of morbidity and mortality (Kellum et al., 2021). It has multiple etiology, such as renal ischemia, nephrotoxic drugs, sepsis, and urinary tract obstruction, and its pathogenesis is also related to various pathways (Bellomo et al., 2012). Nephrotoxic drugs include antibiotics, antipyretic analgesics, chemotherapy drugs, and antiepileptic drugs (Marley et al., 2022). Cisplatin was first approved as an anti-tumor drug in 1978, and it remains an essential and influential therapy for the treatment of various cancers. About 20% of chemotherapy regimens for malignant tumors contain cisplatin (Manohar and Leung, 2018). However, its clinical application is limited due to toxic side effects on normal tissues such as kidney, liver, heart, and nerves (Qi et al., 2019). In addition, the toxicity of cisplatin is positively related to the dose (Dasari and Bernard Tchounwou, 2014). Nephrotoxicity is the main complication, and AKI occurs in about 20%–30% of patients. The primary purpose of treating renal injury by cisplatin is its cause and symptoms. In clinical practice, hydration is often used before and after chemotherapy to promote the excretion of cisplatin to prevent nephrotoxicity. Amifostine is approved by the U.S. Food and Drug Administration for use in reducing progressive nephrotoxicity of repeated cisplatin dosing in patients with advanced ovarian cancer (el Arabey and Abd Allah, 2015). However, the final treatment chiefly relies on renal replacement therapy. Specific drugs are still lacking. Therefore, it is essential to discover effective medications to prevent and cure cisplatin-induced AKI. In ancient Chinese medicine books, AKI belongs to the categories of “edema” and “guange”. The onset is due to a lack of righteousness, exogenous damp-heat toxins, or drug poisoning. AKI caused by cisplatin is due to a lack of vital qi caused by drug poisoning (Li et al., 2019; Liu et al., 2021; Cai et al., 2022). Currently, clinical drugs are lacking for treating renal injury induced by cisplatin (Holditch et al., 2019).

San-Huang decoction (SHD) was the topic of this research. According to the theory of traditional Chinese medicine (TCM), it was found that astragalus, rhubarb, and Sanqi are used in the clinical treatment of AKI and CKD due to drug poisoning (Han et al., 2017; Tian et al., 2020; Chen et al., 2022). Astragalus, rhubarb, and sanqi are derived from *Shen Nong’s Materia Medica*. The three medicines complement each other. Among them, astragalus can replenish the kidney and qi, which is the foundation of nature; Rhubarb can boost blood circulation and eliminate blood stasis, dredge the fu organs and remove turbidity, clear heat, and detoxify; Sanqi promotes blood circulation, dissipates blood stasis and settles pain; All kinds of medicines play a role in strengthening the body, supplementing qi and deficiency, promoting blood circulation and removing blood stasis, clearing the five internal organs, removing turbidity and detoxification. Studies have shown that these three herbs have sound preventive effects against chronic renal insufficiency (Chen et al., 2022), diabetic nephropathy (Han et al., 2017), and AKI (Tian et al., 2020). Astragalus decoction regulates diabetic nephropathy engendered by streptozotocin in rats *via* TGF-β/MAPK/PPAR-γ signaling pathway (Han et al., 2017). A nationwide cohort study found that astragalus and rhubarb provide renal protection and survival benefits in patients with advanced chronic kidney disease (Chen et al., 2022). Sanqi oral liquid enhanced autophagy by reducing apoptosis and ameliorating renal ischemia/reperfusion injury by engaging the ERK/mTOR pathway (Tian et al., 2020). However, the role of SHD in the prevention and treatment of cisplatin-induced AKI and its specific molecular mechanism remain unclear.

Network pharmacology (Nogales et al., 2022) is a systems biology-based discipline that analyzes the targets of various drugs, components, and diseases. Molecular docking (Forli et al., 2016) is the docking of small molecules and protein binding sites. It is widely used to study biomolecular interactions and mechanisms and is applied to structure-based drug design. As a new drug research method, network pharmacology has the characteristics of high efficiency, multiple targets, and convenience. It is now extensively implemented in the research of molecular drug mechanisms and molecular docking. TCM is a great treasure house of the Chinese nation with a history of thousands of years. However, the single and compound components of TCM are complex, and the mechanism of their treatment of diseases is yet imprecise. Applying TCM research in network pharmacology helps explore the specific mechanism of TCM in treating conditions (Li and Zhang, 2013). Relevant studies (Qin et al., 2020; Tian et al., 2020) have used this research method to analyze the active components and explore the therapeutic mechanism of astragalus, rhubarb, and Sanqi. These studies offer a considerable possibility that, through network pharmacology and molecular docking analysis, the potential therapeutic targets of SHD can be further explored, which can help develop clinical strategies for cisplatin-induced AKI.

**FIGURE 1:**
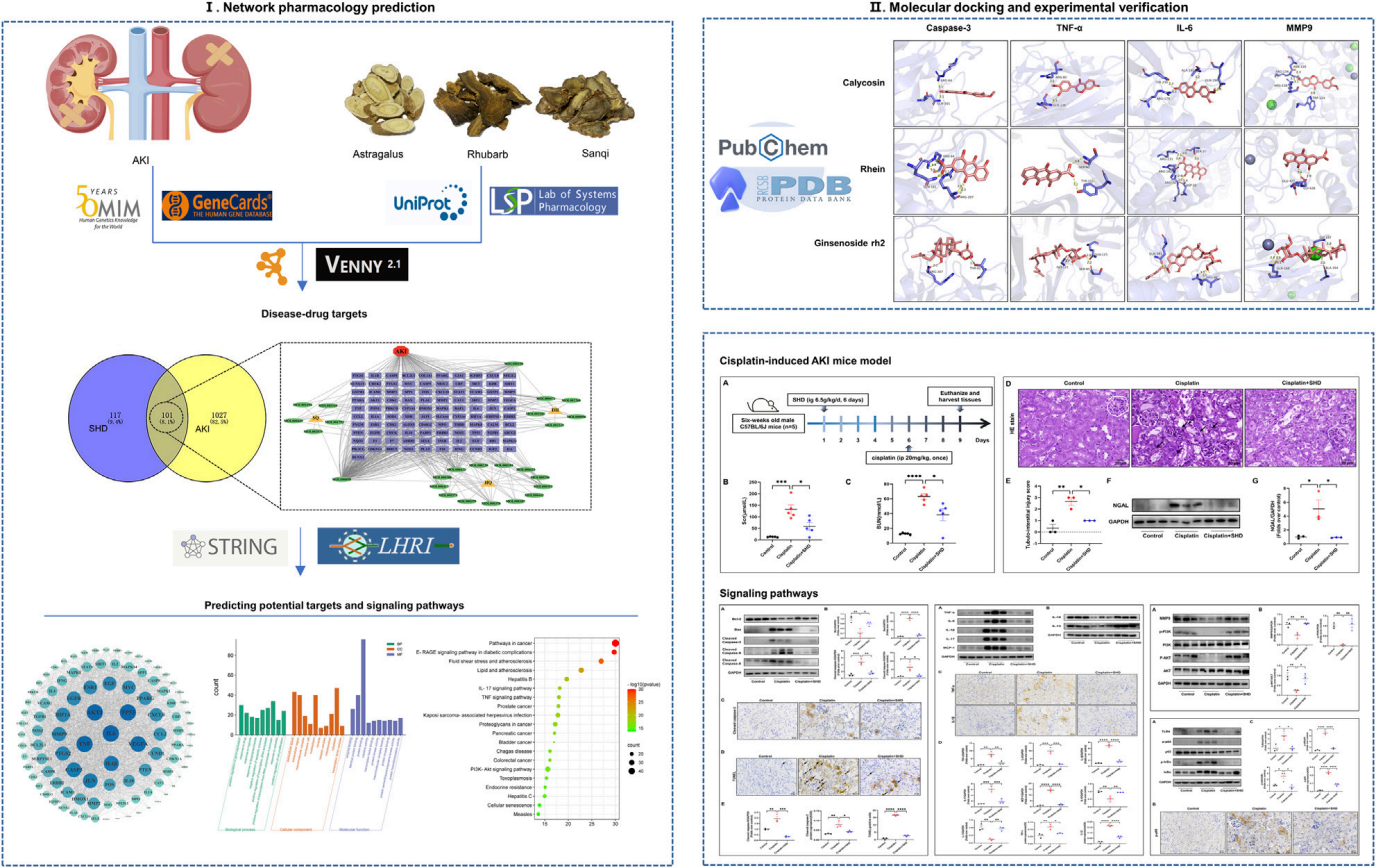
Workflow chart of the research.

In this study, the combined methods of network pharmacology, molecular docking, and experimental verification were used to systematically study the bioactive components of SHD and the mechanism of prevention and treatment of AKI to provide a reference for further research in the future. The workflow of this study is shown in Figure 1.

## Materials and methods

### Network pharmacology analysis

In this study, Traditional Chinese Medicine Systems Pharmacology (TCMSP) database (http://tcmspw.com/tcmsp.php) (Ru et al., 2014) was utilized to screen the most active ingredients and collect component targets of SHD using “Astragalus,” “Rhubarb,” and “Sanqi” as keywords. AKI-related target genes were obtained from Online Mendelian Inheritance in Man (OMIM) (http://www.omim.org) and the GeneCards (https://www.genecards.org/) (Safran et al., 2021) databases. Target and gene names were converted through the Uniprot database (https://www.uniprot.org/) (Bateman et al., 2021). The drug-disease intersection targets obtained from Venny 2.1 (https://bioinfogp.cnb.csic.es/tools/venny/index.html) were fed to the Cytoscape software to draw a network diagram of “drug-component-target-disease” interactions. The intersection targets were potential targets of SHD in preventing and treating cisplatin-induced AKI. The intersection targets were imported into STRING platform (https://string-db.org/) (Szklarczyk et al., 2021), and protein-protein interaction (PPI) was used for association analysis using the network topology analysis *via* CytoNCA plug-in of Cytoscape 3.9.0. The core nodes of the network were then filtered to simplify the network. The intersection targets were imported into DAVID database (https://david.ncifcrf.gov/) (Huang et al., 2009) for Gene Ontology (GO) enrichment analysis and Kyoto Encyclopedia of Genes and Genomes (KEGG) pathway analysis. Enrichment results were then exported as histograms and bubble charts.

### Molecular docking technology

Molecular docking of selected active ingredients and hub genes was performed to validate the accuracy of the target prediction results. PubChem IDs of calycosin, rhein, and ginsenoside Rh2 were retrieved from PubChem database (https://pubchem.ncbi.nlm.nih.gov/) (Kim et al., 2021). Chem3D software was used in the energy minimization process, and AutoDock (http://autodock.scripps.edu) software was utilized to handle molecular cyclic peptides. The 3D protein structures of TNF-a, IL-6, caspase-3, and MMP9 were downloaded from Protein Data Bank (PDB) database (http://www.rcsb.org/pdb/home/home.do), the water molecules and The original small molecule ligand of the receptor protein were removed, adding non-polar hydrogen and charge. Molecular docking of the screened chemical components with the target was performed using AutoDock Vina software.

### Reagents and antibodies

Cisplatin (Lot No. P4394) was from Sigma Aldrich; Hematoxylin-eosin (HE) staining kit (Lot No. C0105), terminal deoxynucleotidyl transferase-mediated nick end labeling (TUNEL) apoptosis detection reagent Box (Lot No. C1098), Protease and phosphatase inhibitor cocktail for general use (Lot No. P1048) and 5 × protein loading buffer (Lot No. P1040) were from Beyotime; Radio-immunoprecipitation assay (RIPA) lysis buffer (Lot No.89900) and bicinchoninic acid assay (BCA) (Lot No. 23227) were from Thermo Fisher; Enhanced chemiluminescence (ECL) reagent (Lot No. WBKLS0500) was from Millipore; Xylene (Lot No. X820585) and absolute ethanol (Lot No. E809056) were from McLean; Goat anti-rabbit IgG (Lot No. ab6721), goat anti-mouse IgG (Lot No. ab6789), anti-NF-kB p65 (Lot No. ab16502), anti-MMP2 (Lot No. ab86607), Anti-Lipocalin-2/NGAL (Lot No. ab41105), and anti-IL-10 (Lot No. ab34843) were from Abcam; Anti-TLR4 (Lot No. #14358), anti-phospho-NF-κB p65 (Ser536) (93H1) (Lot No. #3033), anti-Phospho-IkBa (Lot No. #2859), anti-IκBα (L35A5) (Lot No. #4814), anti-Bax (Lot No. #2772), anti-Caspase-8 (D35G2) (Lot No.#4790), anti-Caspase-9 (C9) (Lot No.#9508), anti-Cleaved-caspase-3 (Asp175) (5A1E) (Lot No.#9664), anti-PI3 Kinase p110α (C73F8) (Lot No. #4249) , anti-Phospho-PI3 Kinase p85 (Tyr458)/p55 (Tyr199)(Lot No. #4228), anti- Akt (pan) (11E7) (Lot No.#4685), and anti-GAPDH (Lot No. #2118) were brought from Cell Signaling Technology (CST); Anti-TNF-α (Lot No. sc-52746), anti-IL-17 (Lot No. sc-374218), anti-IL-6 (Lot No. 373708), anti-IL-1β (Lot No. sc-52012), anti-MCP-1 (Lot No. sc-52701) were from Santa Cruz; Anti-MMP9 (Lot No. P4394) anti-IL13 (Lot No.11059-1-AP) and anti-Bcl2(Lot No. 26593-1-AP) were from Proteintech (Lot No. 10375-2-AP). Anti-Akt (phosphor Ser473) (Lot No. YP0006) was from ImmunoWay. MaxVision HRP-polymer anti-mouse/rabbit secondary antibody (Lot No. KIT-5020), DAB visualization kit (Lot No. DAB-0031), and 10% w/v normal goat serum (Lot No. SP Kit-B2) were from Maixin Biotech.

### Preparation of SHD concentrate

SHD containing astragalus [root, Astragalus membranaceus (Fisch.) Bge. Huangqi], rhubarb [root and rhizome, Rheum palmatum L, Dahuang], and Sanqi [root, Panax notoginseng (Burk.) F. H. Chen, Sanqi], was obtained from Shenzhen Traditional Chinese Medicine Hospital with the dry weight of the product ratio of 2:2:1. Herbs were boiled twice in ddH_2_O for 1 h each. The extraction liquid of SHD was centrifuged, and the supernatant was 450 mg/mL. The concentrated solution was stored at −80°C until subsequent use.

### Animals

All the animal assays were endorsed by the Institutional Animal Care and Use Committee (approved number: TOP-IACUC-2021-0119). Male C57BL/6J mice (6 weeks of age, weighing approximately 18–20 g) were obtained from Shenzhen Topbiotech Co., Ltd. (Shenzhen, China). In typical laboratory conditions, 12 h of darkness and 12 h of light were observed and food and water were freely available to the mice. After a week of adaptive feeding, mice were randomly divided into three groups (*n* = 5): control, Cisplatin, and Cisplatin + SHD. AKI models were established with a single intraperitoneal (i.p.) injection of cisplatin at 20 mg/kg (Chen et al., 2021). 6.5 g of SHD/kg/day were infused for six consecutive days on the Cisplatin + SHD group. Cisplatin and Cisplatin + SHD groups were given i.p. injection of one dose of cisplatin on day six. The control group was i.p. injected with the exact vehicle. After 72 h of cisplatin injection, all mice were sacrificed. The blood and kidney tissues were snap-frozen in liquid nitrogen instantly and then stored at −80°C for further study. Figure 5A depicts the experimental procedure.

### Measurement of Serum

Blood samples were centrifuged at 3,500 rpm for 15 min, and serum was collected for determination. Levels of serum creatinine (Scr) and blood urea nitrogen (BUN) were measured by enzymatic methods (Hitachi cobas 8000; Roche Diagnostics, Germany).

### Hematoxylin and eosin staining

Renal tissue samples were soaked in 10% neutral buffered formalin overnight. Tissue samples fixed in formalin were dehydrated in graded alcohols (100%, 95%, 85%, and 75%) and embedded in paraffin wax. They were cut into 4–5 μm sections. Hematoxylin and eosin (H&E) staining was done according to the kit protocols to assess tubular injury conditions and kidney morphological alterations. H&E sections were evaluated and scored according to renal cortical vacuolization, infiltration of peritubular and proximal tubule leukocytes, and simplification of the proximal tubules: 0, normal; 1, mild injury; 2, moderate injury; 3, severe injury (Chang et al., 2016).

### Western blot

Frozen kidney tissue samples were pulverized by a high-throughput tissue grinder and then lysed in RIPA buffer containing protease and phosphatase inhibitors. Proteins were determined using the BCA. An electrophoresis with sodium dodecyl sulfate-polyacrylamide gel (SDS-PAGE) was performed with 10% or 15% gels and 70-min runs at 120 mV. The proteins were then transferred to polyvinylidene fluoride (PVDF) membranes. After blocking with 5% non-fat milk for 2 h, the membrane was incubated with primary antibody overnight at 4°C. The dilution ratio of Abcam, CST, and proteintech primary antibody products was 1:1000, and the dilution ratio of Santa Cruz primary antibody products was 1:250. The membranes were then incubated with horseradish peroxidase (HRP)-conjugated secondary antibodies and diluted 2000-fold in 5% skimmed milk for 1 h. Protein bands were detected with chemiluminescent HRP substrate, and band intensities were calculated with ImageJ software.

### Immunohistochemistry

The sections of paraffin-embedded renal tissues of mice were allowed to dry 1 h before immunohistochemistry (IHC) staining. We dewaxed the sections, placed them in boiling sodium citrate buffer for 20 min, and then immersed them for 15 min in 3% H_2_O_2_. Following phosphate-buffered saline (PBS) washing, 10% goat serum was incubated at room temperature for 15 min. To bind specifically to primary antibodies, the sections were hatched for 12–14 h at 4°C with the primary antibodies, including TNF-α (1:500), IL-1β (1:200), cleaved-caspase-3 (1:1200), and phospho-NF-κB p65 (1:200). Sections were allowed to return to room temperature for at least 30 min. After treating with HRP-Polymer anti-Mouse/Rabbit for 15 min, the sections were placed under the microscope, and their color change was detected with diaminobenzidine (DAB) chromogenic solution. Hematoxylin was used in counterstaining. Images were acquired using an optical microscope (Carl Zeiss, Germany). Kidney tissue pictures from all groups were randomly taken in three equal microscopic fields (×400). Quantitative analysis was operated using ImageJ software.

### TUNEL staining

TUNEL staining for formalin-fixed renal sections was conducted using the one-step TUNEL apoptosis assay kit with a colorimetric assay. Deparaffinization and washing were the same as the procedures of H&E. Sections were allowed to incubate with proteinase K (20 μg/mL) for 20 min at room temperature and rinsed with PBS. All sections were incubated with hydrogen peroxide to inhibit endogenous peroxidase for 20 min. A 50*l TUNEL reaction solution (enzyme: marking liquid = 1:9) was added to each section according to TUNEL detection kit instructions. Each section was washed for 5 min with PBS three times. After staining with DAB, they were examined under a microscope. Three different fields of view (×400) for each group were randomly selected for TUNEL-positive cell counts.

### Statistical analysis

Statistics were performed on all data using GraphPad Prism 9.0 software. Each data group had at least three independent samples, the two-tailed Student's t-test was used to compare the two groups, and one-way analysis of variance was exploited to evaluate the differences between multiple groups. Data are expressed as mean ± SEM with a significant level of *p* < 0.05.

## Results

### Network pharmacology analysis results

#### Analysis of active ingredients of SHD

SHD is composed of astragalus, rhubarb, and Sanqi. Through TCMSP, compounds with bioavailability ≥30% and drug-like properties ≥0.18 were selected as candidate components. The active component targets were screened and analyzed using the Swiss Target Prediction and Uniprot databases. After the targets were normalized and the duplicate values were deleted, 117 final targets of SHD and 25 corresponding active compounds (two overlapped) were obtained (Table 1).

**TABLE 1:** Information of active components of San-Huang decoction.

Number	Molecule name	OB (%)	DL	Molecules structure	Herb
MOL002281	Toralactone	46.46	0.24	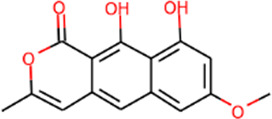	Rhubarb
MOL002268	Rhein	47.07	0.28	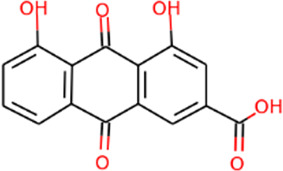	Rhubarb
MOL002235	Eupatin	50.80	0.41	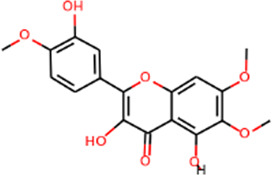	Rhubarb
MOL000471	Aloe-emodin	83.38	0.24	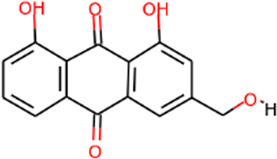	Rhubarb
MOL000358	Beta-sitosterol	36.91	0.75	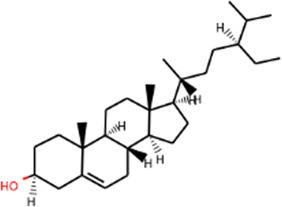	Rhubarb/Sanqi
MOL000096	(−)-Catechin	49.68	0.24	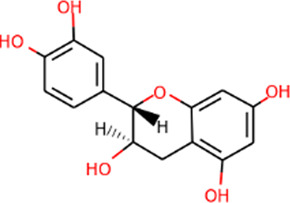	Rhubarb
MOL000442	1,7-Dihydroxy-3,9-dimethoxy pterocarpene	39.05	0.48	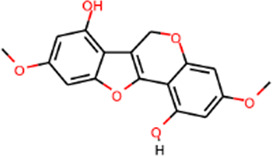	Astragalus
MOL000433	FA	68.96	0.71	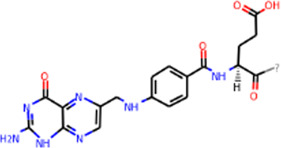	Astragalus
MOL000422	Kaempferol	41.88	0.24	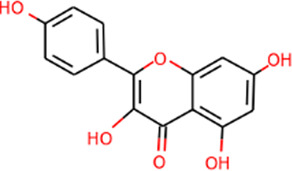	Astragalus
MOL000417	Calycosin	47.75	0.24	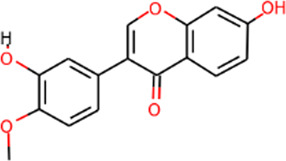	Astragalus
MOL000392	Formononetin	69.67	0.21	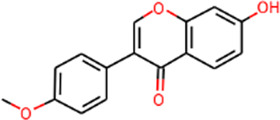	Astragalus
MOL000387	Bifendate	31.10	0.67	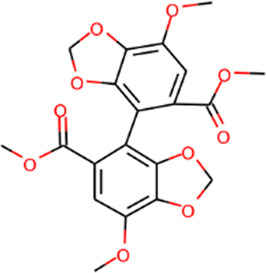	Astragalus
MOL000380	(6aR,11aR)-9,10-Dimethoxy-6a,11a-dihydro-6H-benzofurano [3,2-c]chromen-3-ol	64.26	0.42	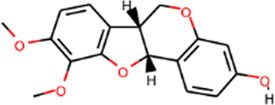	Astragalus
MOL000379	9,10-Dimethoxypterocarpan-3-O-β-D-glucoside	36.74	0.92	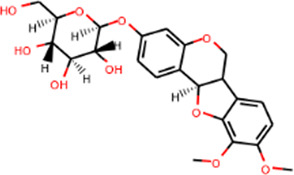	Astragalus
MOL000378	7-O-Methylisomucronulatol	74.69	0.30	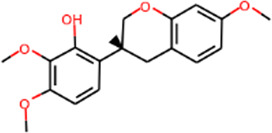	Astragalus
MOL000371	3,9-di-O-methylnissolin	53.74	0.48	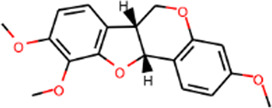	Astragalus
MOL000354	Isorhamnetin	49.60	0.31	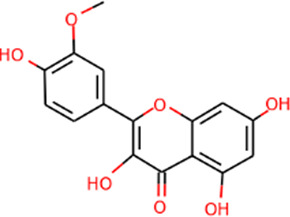	Astragalus
MOL000296	Hederagenin	36.91	0.75	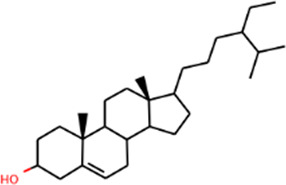	Astragalus
MOL000239	Jaranol	50.83	0.29	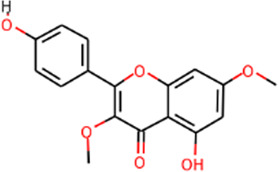	Astragalus
MOL000098	Quercetin	46.43	0.28	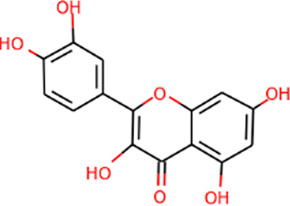	Astragalus/Sanqi
MOL005344	Ginsenoside rh2	36.32	0.56	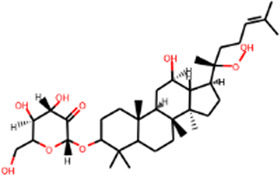	Sanqi
MOL002879	Diop	43.59	0.39	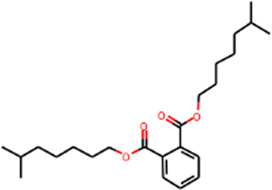	Sanqi
MOL001792	DFV	32.76	0.18	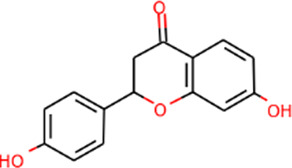	Sanqi
MOL001494	Mandenol	42.00	0.19	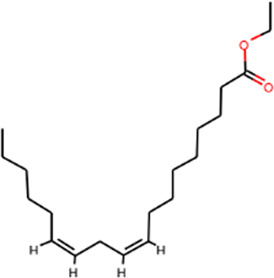	Sanqi
MOL000449	Stigmasterol	43.83	0.76	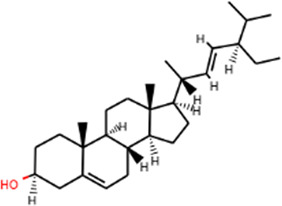	Sanqi

#### SHD and cisplatin-induced AKI targets

**FIGURE 2:**
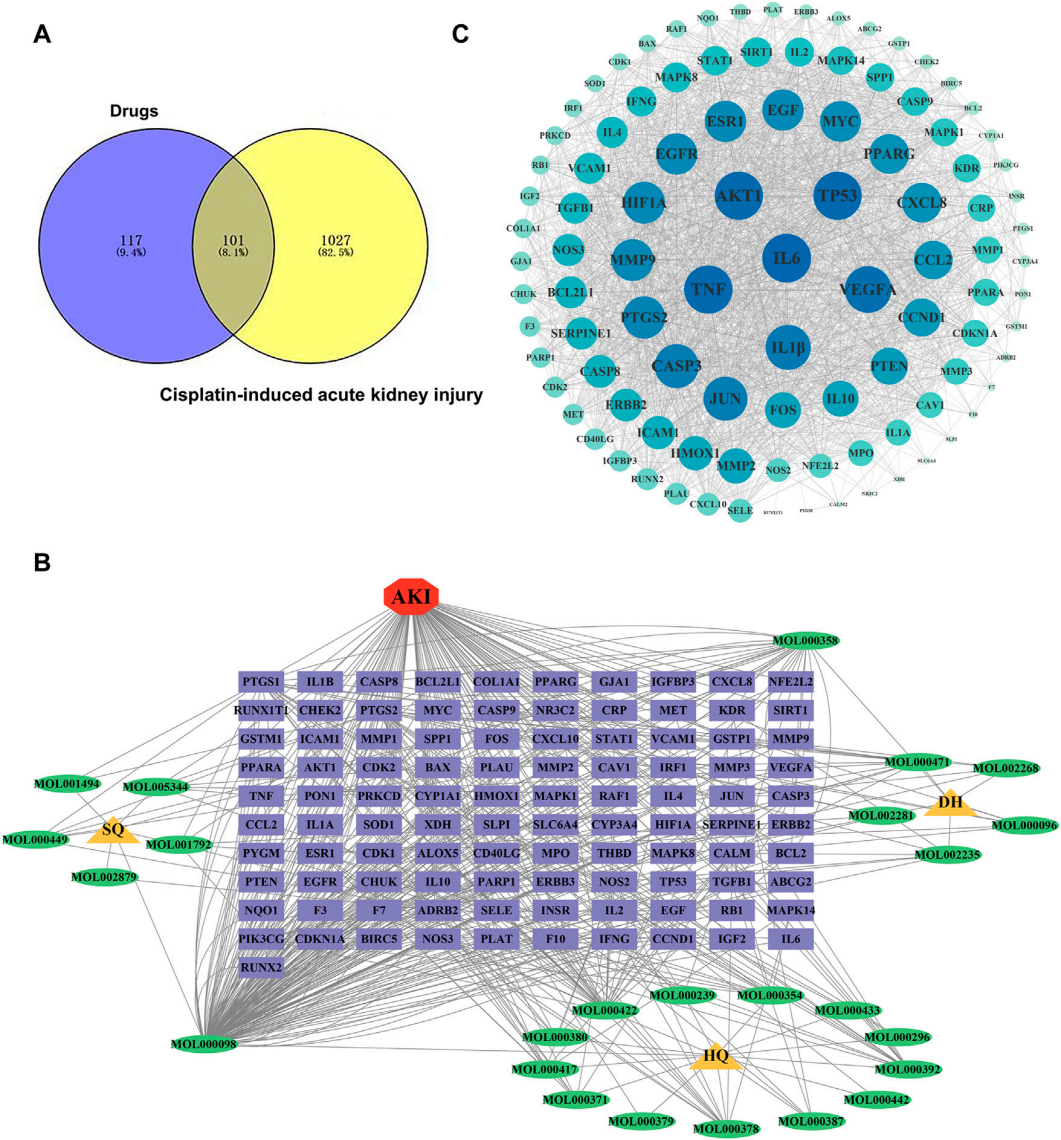
Identification of potential targets-active ingredients network. **(A)** Venn diagram analysis of SHD and cisplatin-induced AKI. **(B)** Drug-composition-target-disease of SHD-cisplatin-induced AKI. HQ is the abbreviation of astragalus. DH is the abbreviation of rhubarb. SQ is the abbreviation of sanqi. **(C)** Target PPI network of SHD-cisplatin-induced AKI.

By searching “Cisplatin-induced nephrotoxicity, Cisplatin-induced renal toxicity, and Cisplatin-induced acute kidney injury” as keywords, we identified 157 AKI-related targets from OMIM and GeneCards databases, and 1027 AKI targets were retrieved from the GeneCards and OMIM databases. AKI target information was put into the online Venny 2.1 platform for Venn analysis, and 101 intersecting targets were obtained (Figure 2A).

#### Constructing a network of “TCM-ingredients-targets-disease”

Following the intersection, the target of “Astragalus-Rhubarb-Sanqi” in treating cisplatin-induced AKI was obtained and entered into Cytoscape software. The interaction of “TCM-ingredient-target-disease” was drawn in the network diagram (Figure 2B).

#### PPI network analysis of SHD and cisplatin-induced AKI

The typical target genes of SHD and cisplatin-induced AKI were entered into STRING database to obtain protein interaction data information and then imported into Cytoscape software to draw PPI network. A total of 102 nodes (target genes) in PPI network interacted through edges (Figure 2C). The protein network structure was visualized with Cytoscape software according to the degree. The outcome of PPI analysis showed that the critical target genes of SHD-cisplatin-induced AKI were AKT, MMP9, IL-6, TNF-α, IL-1β, and caspase-3.

#### GO and KEGG enrichment analyses

**FIGURE 3:**
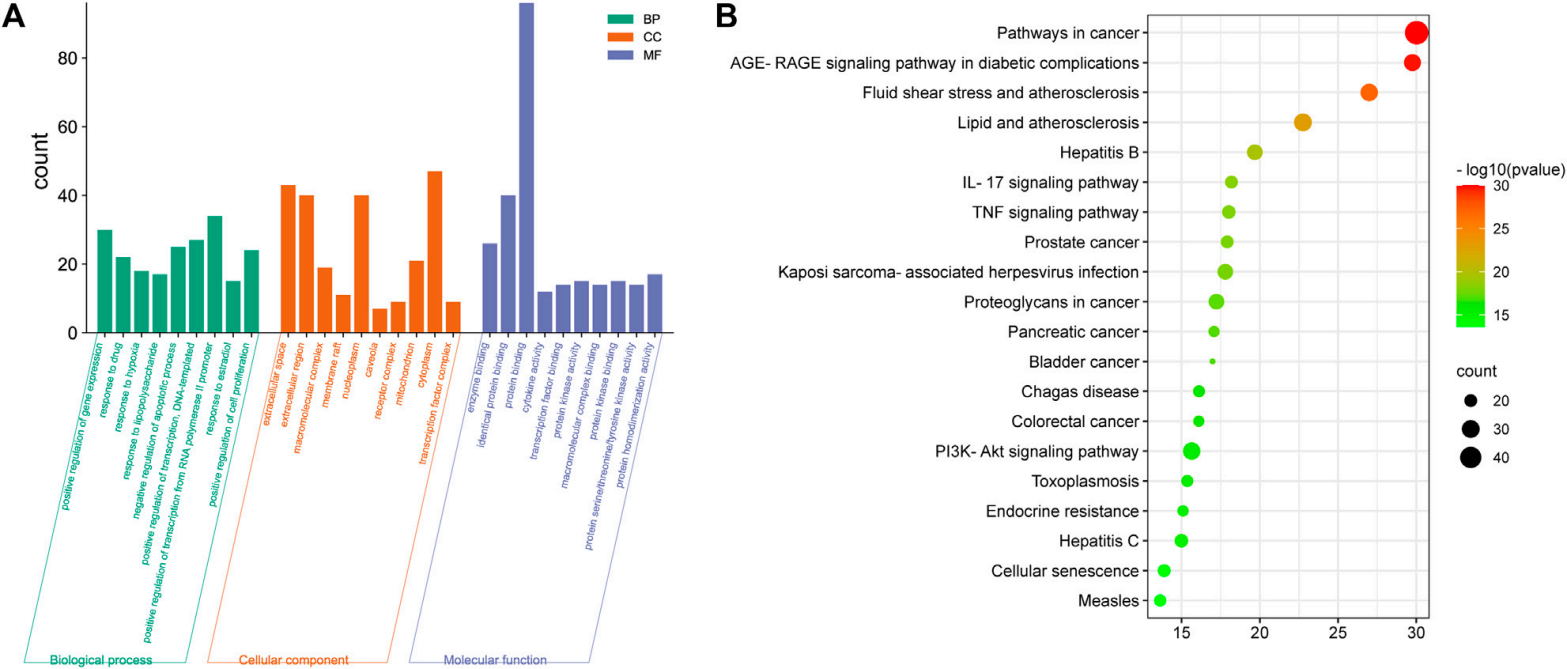
Functional enrichment analysis of targets. **(A)** GO enrichment analysis and **(B)** KEGG pathway enrichment analysis of SH-cisplatin-induced AKI.

To elucidate the biological processes involved in SHD-cisplatin-induced AKI targets, KEGG pathway and GO functional enrichment analyses were applied on the targets using DAVID database. GO functional enrichment analysis revealed that 628 biological processes (BPs), 63 cellular components (CCs), and 124 molecular functions (MFs) were enriched. Figure 3A showed the top 10 results in BPs, CCs, and MFs. The results indicated the following biological processes: cytokine-mediated signaling, positive regulation of gene expression, response to drugs, positive regulation of RNA polymerase II promoter transcription, negative regulation of apoptotic processes, lack of exogenous ligands, apoptosis signaling pathway, and inflammatory response. KEGG pathway enrichment analysis showed that 157 pathways were enriched, and the first 20 KEGG analysis results were screened with *p* < 0.05 as the threshold (*p* < 0.01, gene frequency >0.05%). The results included IL-17 signaling pathway, TNF-α signaling pathway, etc. (Figure 3B).

#### “Active ingredient-target” molecular docking

**FIGURE 4:**
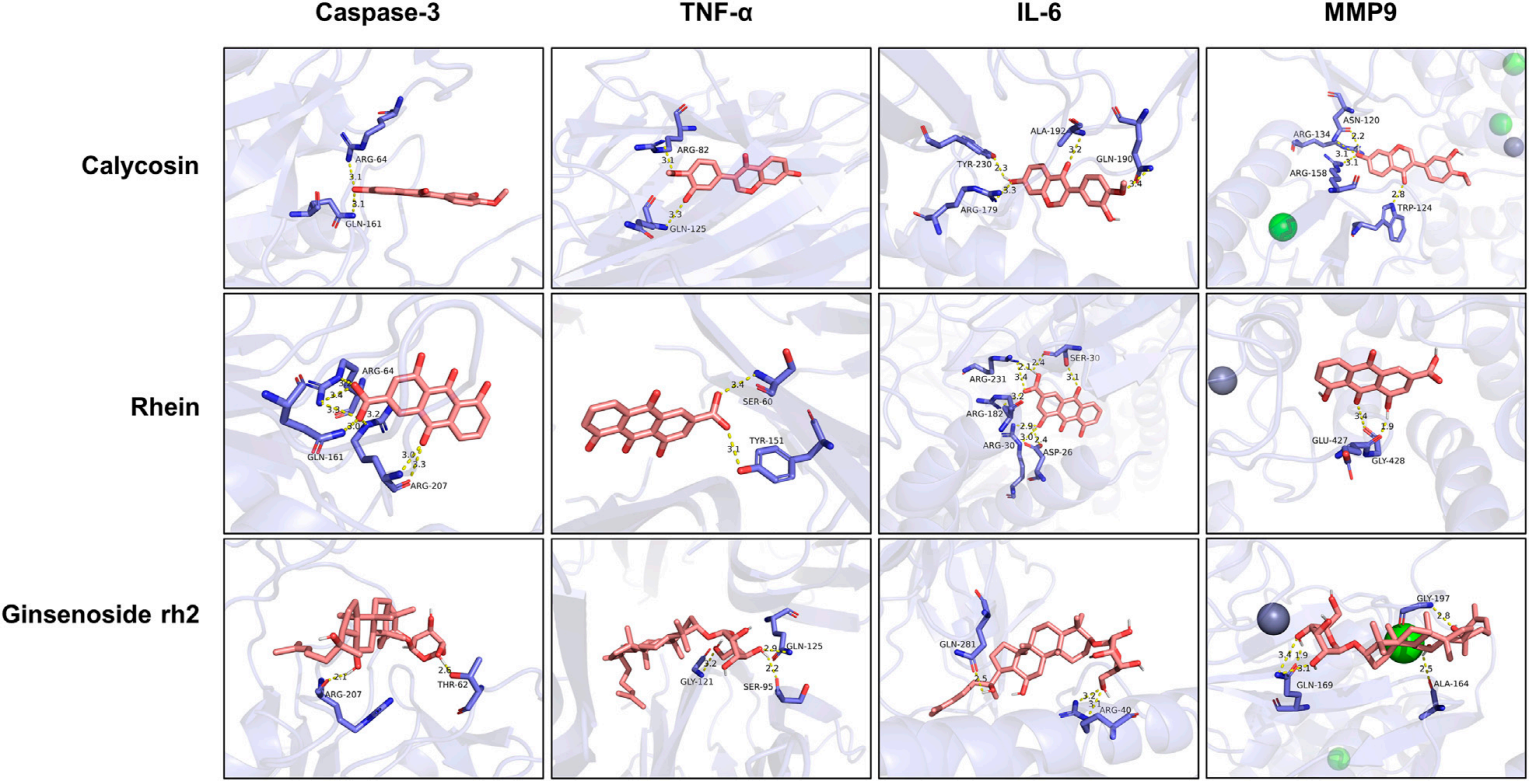
The molecular docking results between active ingredients and hub targets.

The three main active compounds important in SHD are calycosin, rhein, and ginsenoside Rh2. Their molecular docking and binding abilities to key targets TNF-α, IL-6, caspase-3, and MMP9 were predicted. The smaller the free binding energy, the greater the affinity between the receptor and the ligand. Three main active ingredients and key target proteins showed binding free energies below −7 kJ/mol (Figure 4), indicating that the active ingredients had a high affinity with the target protein. Among them, the affinity of TNF-α and ginsenoside Rh2 was the highest. The affinity of IL-6 and rhein was the highest (Table 2).

**TABLE 2:** Molecular docking results between ligands and core target receptors.

Target genes	UniProt id	Active ingredients	PubChem CID	Binding energy (kcal/mol)
TNF-α	P01375	Calycosin	5,280,448	−8.7
TNF-α	P01375	Rhein	10,168	−8.4
TNF-α	P01375	Ginsenoside rh2	119,307	−9.2
IL-6	P05231	Calycosin	5,280,448	−7.6
IL-6	P05231	Rhein	10,168	−9.2
IL-6	P05231	Ginsenoside rh2	119,307	−8.5
Caspase-3	P42574	Calycosin	5,280,448	−8.1
Caspase-3	P42574	Rhein	10,168	−8.0
Caspase-3	P42574	Ginsenoside rh2	119,307	−8.7
MMP9	P14780	Calycosin	5,280,448	−7.4
MMP9	P14780	Rhein	10,168	−7.8
MMP9	P14780	Ginsenoside rh2	119,307	−7.9

### Experimental verification of SHD preventing and treating cisplatin-induced AKI in C57BL/6J mice

#### SHD can alleviate cisplatin-induced AKI in mice

**FIGURE 5:**
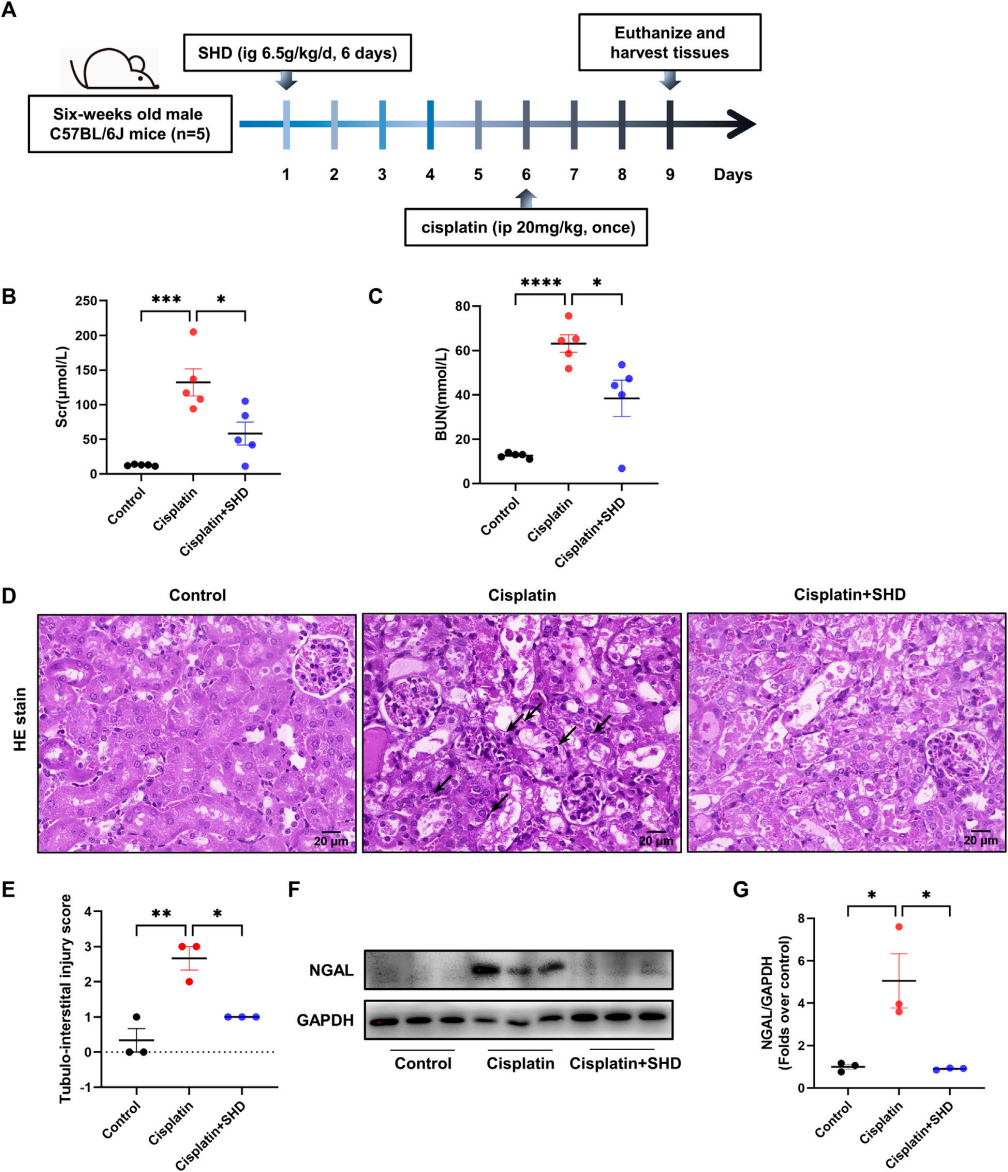
Effects of SHD on renal dysfunction and tubular damage induced by cisplatin. **(A)** Diagram of the experimental protocol. **(B)** Serum levels of creatinine and **(C)** blood urea nitrogen levels were measured (*n* = 5). **(D)** Representative kidney section stained for hematoxylin and eosin in each mice group. Arrowheads indicate inflammatory cells or cell debris. **(E)** Represents the semiquantitative analysis of tubule interstitial injury. Representative kidney section stained for hematoxylin and eosin in each mice group. Arrowheads indicate inflammatory cells or cell debris. Scale bar = 20 μm. Original magnification ×400. **(F)** Representative Western blot images of NGAL in the kidneys. **(G)** Densitometric analyses of NGAL proteins expression normalized to GAPDH content (*n* = 3). Data presented are means ± SEM. *p < 0.05, **p < 0.01.

To verify that SHD has a therapeutic effect on cisplatin-induced AKI, *in vivo* animal experiments were carried out. After 72-h cisplatin injection, the animals showed significant body weight loss, while their kidney weight increased significantly along with the levels of Scr and BUN. Singularly, SHD significantly reversed the situation. In comparison to the control group, the Scr levels in the cisplatin group was significantly elevated (132.2 μmol/L vs. 12.6 μmol/L, *p* < 0.05). SHD treatment decreased serum creatinine levels in mice with AKI (58.2 μmol/L, *p* < 0.05) (Figure 5B).

As compared to the control group, the BUN level in the cisplatin group was also increased to 61.13 mmol/L (12.6 mmol/L in the control group, *p* < 0.05). SHD treatment reduced blood urea nitrogen levels in mice with AKI (38.39 mmol/L, *p* < 0.05) (Figure 5C). Histologically, compared with the control group, the periglomerular tubules (proximal tubules) in the cisplatin group had obvious lesions (Figures 5D,E). There was a massive infiltration of inflammatory cells in the renal tubular space. With SHD treatment, these renal tissue damages were significantly reversed. Western blot analysis showed that neutrophil gelatinase-associated lipocalin (NGAL), one of the best markers of AKI, was significantly less in kidneys from the cisplatin + SHD group compared to those from the cisplatin group (Figures 5F,G).

#### SHD promotes the activation of the MMP9/PI3K/AKT pathway in kidney tissue of cisplatin-induced AKI mice

**FIGURE 6:**
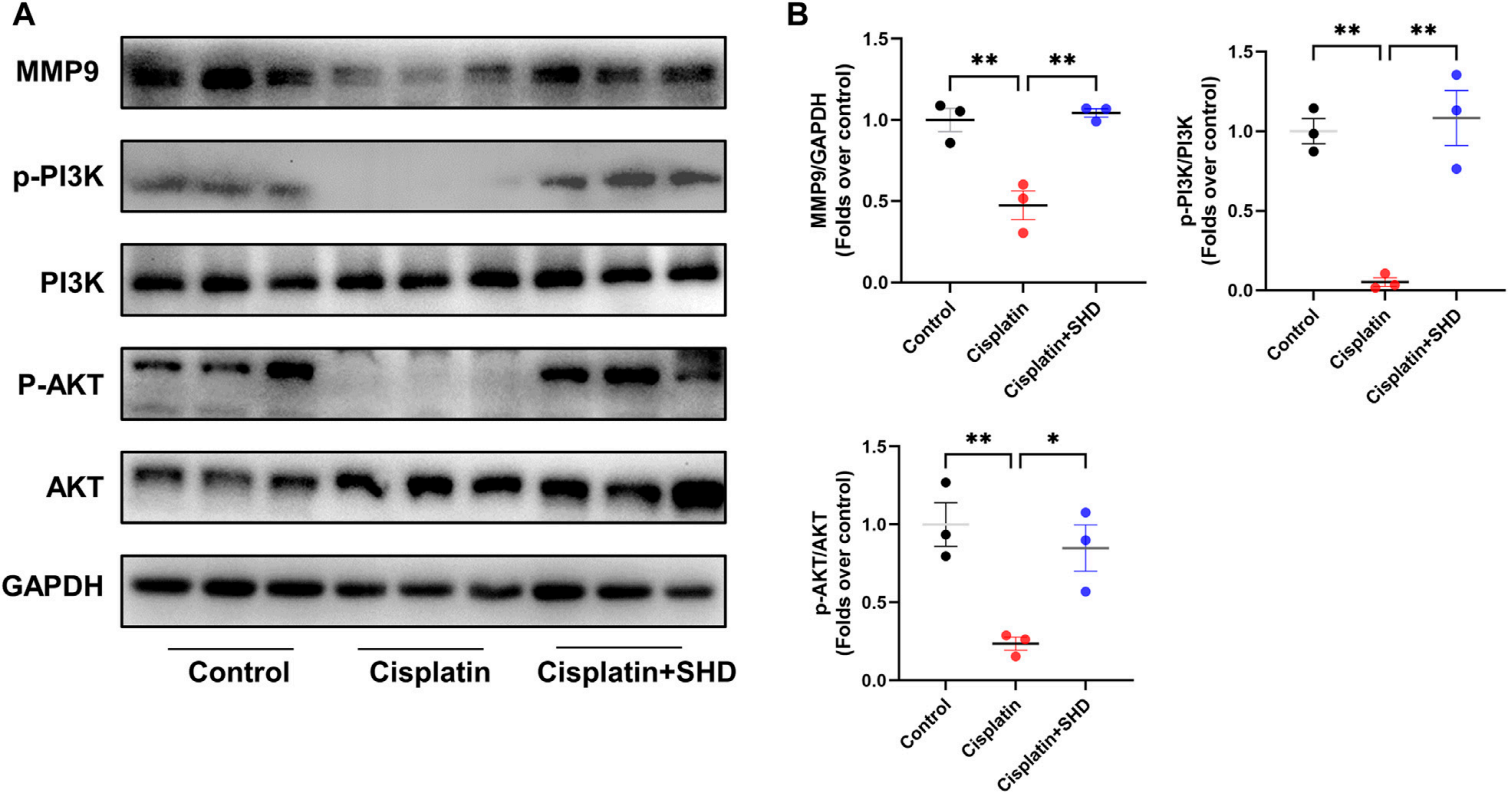
Effects of SHD on MMP9/PI3K/AKT pathway activity in the kidney. **(A)** Representative Western blot images of MMP9, phospho-PI3K, PI3K, phospho-AKT and AKT in the kidneys. **(B)** Densitometric analyses of MMP9, phospho-PI3K/PI3K, phospho-AKT/AKT proteins expression normalized to GAPDH content (*n* = 3). Data presented are means ± SEM. ^∗^ *p* < 0.05, ^∗∗^ *p* < 0.01.

Studies (Wozniak et al., 2021) have shown that a family of MMPs plays a role in AKI and that MMP9 can activate the PI3K/AKT pathway to resist apoptosis. PPI analysis also presented that MMP9 is critical in predicting SHD the prevention and treatment of cisplatin-induced AKI. Consistent with the expected results, it indicated that the activity of MMP9 was decreased in the Cisplatin group (Figure 6). In contrast, the activity of MMP9 was significantly up-regulated in the Cisplatin + SHD group. The PI3K/AKT pathway exhibited the same trend (Figure 6). SHD reversed the decreased MMP9 activity and inhibition of the PI3K/AKT pathway in the Cisplatin group.

#### SHD inhibits the level of apoptosis in kidney tissue of cisplatin-induced AKI mice

**FIGURE 7:**
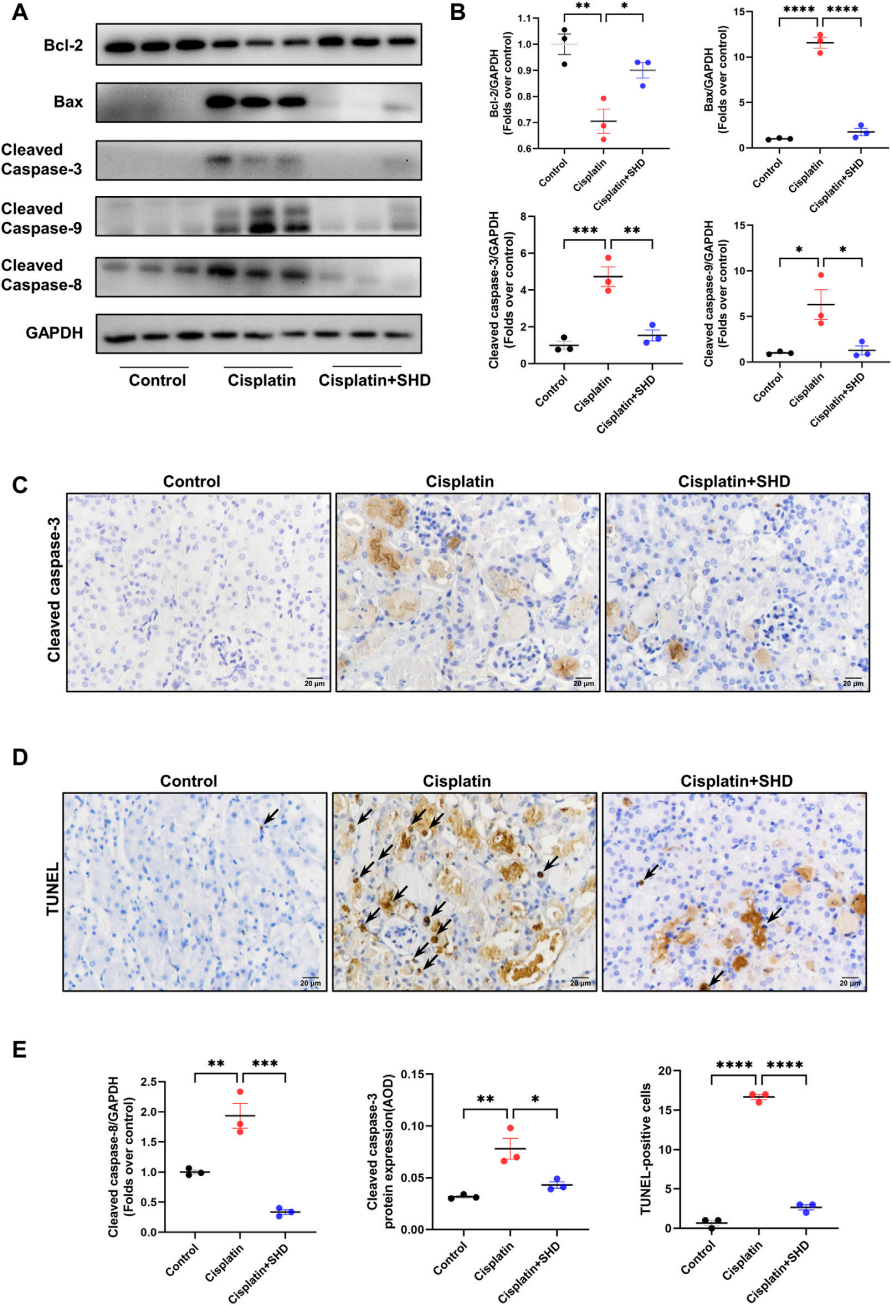
Effects of SHD on apoptosis induced by Cisplatin in the kidney. **(A)** Representative Western blot images of Bcl-2, Bax, Cleaved-Caspase3, Cleaved-Caspase8, and Cleaved-Caspase9 in the kidneys. **(B)** Densitometric analyses of BCl2, Bax, Cleaved-Caspase8, and Cleaved-Caspase9 proteins expression normalized to GAPDH content (*n* = 3). **(C)** Representative immunohistochemistry (IHC) images of Cleaved-Caspase3 protein in renal cortical glomeruli and tubular epithelium (400 ×). Scale bar = 20 μm. **(D)** Representative results of TUNEL staining of the kidney section in each mice group (*n* = 3). Arrowheads indicate TUNEL-positive cells. Scale bar = 20 μm. **(E)** Densitometric analyses of Cleaved-Caspase3 protein expression normalized to GAPDH content (*n* = 3). Semiquantitative analysis of IHC for the Cleaved-Caspase3 protein expression. AOD = IOD/area. Semiquantitative analysis of TUNEL-positive cells. Data presented are means ± SEM. ^∗^ *p* < 0.5, ^∗∗^ *p* < 0.01, ^∗∗∗^ *p* < 0.001, ^∗∗∗∗^ *p* < 0.0001. Original magnification ×400.

Consistent with previous literature (Deng et al., 2020), PPI network and GO functional enrichment analyses showed that the SHD-cisplatin-induced AKI target was related to the critical apoptotic protein caspase-3 and the apoptotic process. Western blotting (Figures 7A,B) and IHC (Figures 7C,E) results showed that compared with the Cisplatin group, SHD treatment significantly reversed the dysregulation of the related proteins Bcl-2, caspase-3, caspase-8, caspase-9, and Bax in the two pathways of apoptosis and improved cisplatin-induced AKI apoptosis in mice. Consistently, we used TUNEL staining to detect the fragmentation of nuclear DNA in mouse tissue cells during apoptosis. In control mouse kidney tissue, there are very few TUNEL-positive cells. Following cisplatin injection, TUNEL-positive cells were markedly increased in mouse kidney tissue, which was significantly blocked after SHD administration (Figures 7D,E).

#### SHD inhibits inflammation in kidney tissue of cisplatin-induced AKI mice

**FIGURE 8:**
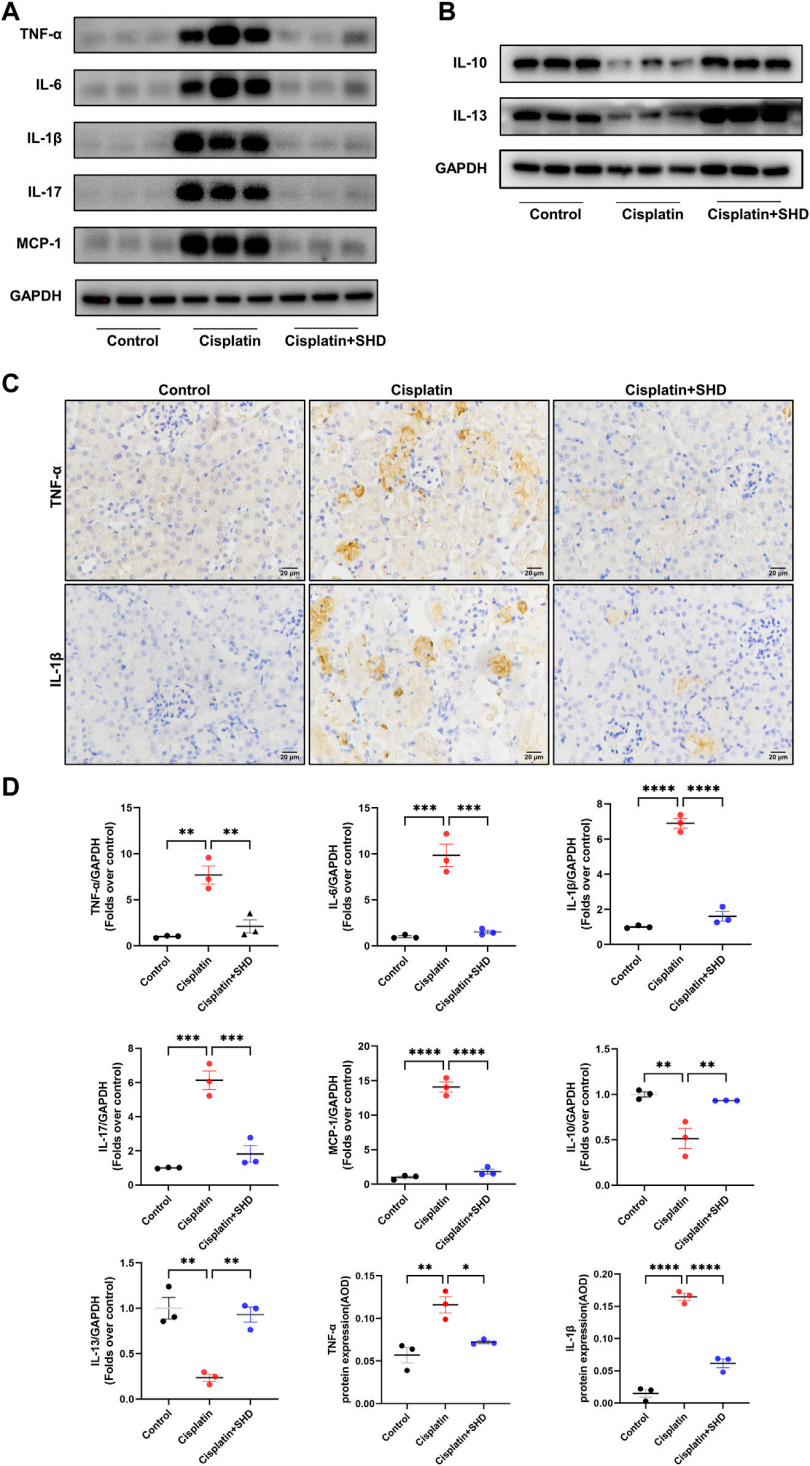
Effects of SHD on inflammation induced by Cisplatin in the kidney. **(A)** Representative Western blot images of pro-inflammatory cytokines, such as TNF-α, IL-6, IL-1β, IL-17, and MCP-1 in the kidneys. **(B)** Representative Western blot images of anti-inflammatory factors, such as IL-13 and IL-10 in the kidneys. **(C)** Representative IHC images of TNF-α and IL-1β protein in renal cortical glomeruli and tubular epithelium. Scale bar = 20 μm **(D)** Densitometric analyses of TNF-α, IL-6, IL-1β, IL-17, MCP-1, IL-10 and IL-13 proteins expression normalized to GAPDH content (*n* = 3). Quantitative analysis of IHC for the TNF-α and IL-1β protein expression. AOD = IOD/area. Data presented are means ± SEM. ^∗^ *p* < 0.5, ^∗∗^ *p* < 0.01, ^∗∗∗^ *p* < 0.001, ^∗∗∗∗^ *p* < 0.0001.

The role of pro-inflammatory and anti-inflammatory cytokines (e.g., TNF-α, IL-6, IL-1β, IL-17, IL-10, and IL-13) in AKI caused by cisplatin has been widely reported (Lei et al., 2021; Zeinali et al., 2022). Monocyte chemoattractant protein 1 (MCP-1) acts as a pro-inflammatory factor, participating in the pathological process of AKI (Ramesh and Reeves, 2002). Previous studies indicate that MCP-1 equals NGAL as a biomarker of AKI due to its high sensitivity and that urinary MCP-1 has prognostic significance for both short-term and long-term kidney function after surgery (Munshi et al., 2011). As revealed in Figure 8, western blot and IHC analysis showed that pro-inflammatory cytokines in the cisplatin group were significantly up-regulated and were strikingly reversed after SHD treatment. Anti-inflammatory cytokines exhibited the opposite trend. These findings indicated the anti-inflammatory effect of SHD in cisplatin-induced AKI.

#### SHD inhibits the activation of the NF-κB signaling pathway in kidney tissue of AKI mice induced by cisplatin

**FIGURE 9:**
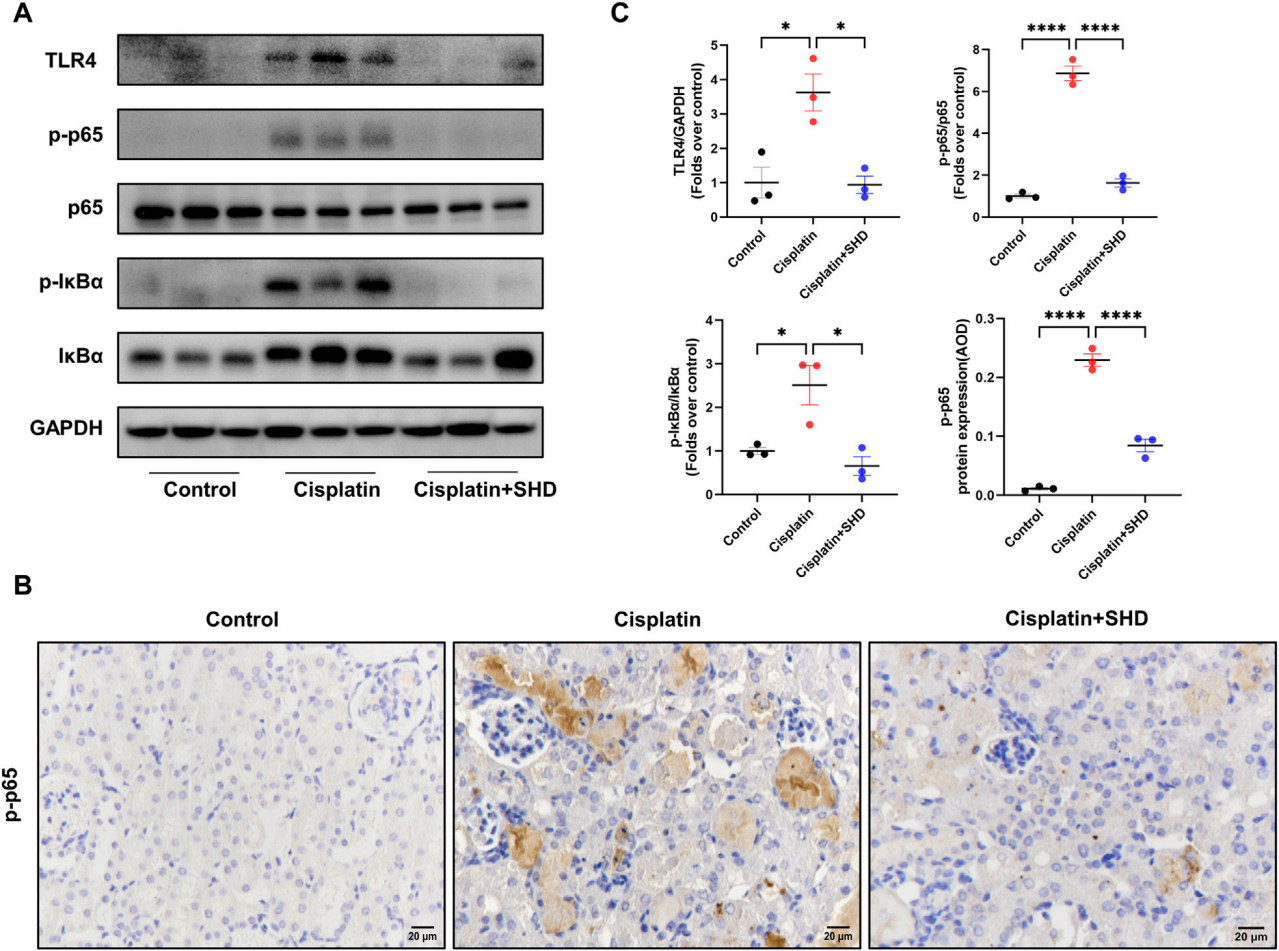
Effects of SHD on NF-κB activity in the kidney. **(A)** Representative Western blot images of TLR4, phospho-NF-κB p65, NF-κB p65, phospho-IkBa, and IkBa in the kidneys. **(B)** Representative IHC images of phospho-NF-κB p65 protein in renal cortical glomeruli and tubular epithelium. Scale bar = 20 μm. **(C)** Densitometric analyses of phospho-NF-κB p65/NF-κB p65, phospho-IkBa/IkBa, and TLR4 proteins expression normalized to GAPDH content (*n* = 3). Semiquantitative analysis of IHC for the phospho-NF-κB p65 protein expression. Data presented are means ± SEM. ^∗^ *p* < 0.5, ^∗∗∗∗^ *p* < 0.0001. Original magnification ×400.

To delve into the protective mechanism of SHD against cisplatin-induced AKI, we further assessed the function of SHD on the activation of the NF-κB signaling pathway in renal tissue. The NF-κB signaling pathway is closely tied with pro-inflammatory cytokines and caspase-3 (Deng et al., 2020). Western blot analysis (Figures 9A,C) showed that phospho-NF-κB p65, phospho-IkBa, and toll-like receptor 4 (TLR4) were significantly up-regulated after cisplatin injection, suggesting that the NF-κB signaling pathway was activated. Likewise, IHC (Figures 9B,C) analysis showed that the protein expressions of phospho-NF-κB p65 in the cisplatin group were higher than those in the control group, which were significantly inhibited by SHD treatment. In our study, NF-κB signaling, inflammation, and apoptosis were all activated in the Cisplatin group.

## Discussion

AKI is a global disease with a poor prognosis. At present, cisplatin-induced AKI can neither be treated nor prevented. Renal replacement therapy becomes necessary as kidney function deteriorates and the disease progresses to its end stage. Consequently, effective treatment for cisplatin-induced nephrotoxicity is urgently needed.

SHD is refined from traditional Chinese prescriptions used for the clinical treatment of various kidney diseases. Astragalus, rhubarb, and Sanqi were recorded in “*Shen Nong’s Materia Medica.*” However, the complex chemical composition makes it difficult to elucidate the pharmacological mechanism of its underlying active compounds and treatment, despite its broad pharmacological activity.

Network pharmacology and molecular docking provide useful methods for the research of the “multi-component, multi-target, multi-biological regulatory function synergy” model of TCM, and have been widely utilized in TCM research and advanced drug findings. In this research, a combination of network pharmacology, molecular docking, and experimental validation was used to systematically investigate the bioactive components of SHD and its therapeutic mechanism for AKI.

Many overlapping targets were found in different compounds in SHD, indicating that SHD exerts anti-inflammatory and anti-apoptotic properties through the synergistic effect of its compounds. The primary active components in SHD may be the key to preventing and treating AKI. Among them, calycosin is the staple active component of astragalus. In studies, it has been shown to reduce renal ischemia-reperfusion injury by inhibiting NF-κB mediated inflammation through the PPARγ/EGR1 pathway (Zhang et al., 2022), and it is anti-inflammatory and promotes angiogenesis. Rhein is the main active component of rhubarb. Consequently, many AKI patients have progressed to chronic kidney disease. Rhein is able to curb the transdifferentiation of renal tubular epithelial cells to myofibroblasts. Studies (Ma et al., 2018) have found that emodin can inhibit the expression of transforming growth factor β1, block the TGF-β1/Smad signaling pathway, and inhibit the transdifferentiation of human renal tubular epithelial cells (HK-2), thereby reducing epithelial-myofibroblast transdifferentiation (TEMT). Rhein and its derivatives, analogs, and compound preparations protect against various kidney diseases, particularly diabetic nephropathy (DN) and drug-induced AKI (Zhu et al., 2022). In the research of Luo Yan et al., Rhein was found to restrain the SHH-Gli1-Snail signal pathway, extenuating renal fibrosis in rhein-treated UUO rats (Luo et al., 2022).

Ginsenoside Rh2 is the main active ingredient of Sanqi (Li et al., 2020). There is evidence that ginsenoside Rh2 has anticancer properties. Cisplatin is a clinically efficient broad-spectrum anticancer drug. Cisplatin nephrotoxicity affects nephrocyte apoptosis. Studies (Qi et al., 2019) have revealed that Rh2 has a protective effect on cisplatin-induced kidney dysfunction and can improve cisplatin-induced renal histopathological damage, inflammatory response, and renal tubular cell apoptosis. In the study of Kim Long Vu Huynh et al. (Vu-Huynh et al., 2019), from processed Vietnamese ginseng, they isolated six PPD-type saponins, including ginsenoside Rh2 and ocotillo genin, that displayed the potential to protect the kidney from cisplatin-induced toxicity. In addition, GO, and KEGG pathway analyses revealed that SHD mainly interferes with AKI occurrence and development through TNF and IL-17 signaling pathways. The abnormal activation of these targets can promote the biological process of AKI, such as inflammation, proliferation, and angiogenesis, further aggravating the condition. Treating tubular epithelial cells is a top priority in AKI. Inflammation, apoptosis, and vascular injury of renal tubular epithelial cells are all important potential targets. Therefore, treating inflammation and regulating apoptosis to reduce vascular injury are the focus of treating AKI. In the results of PPI analysis, we found that AKT, TNF-α, IL-6, IL-1β, caspase-3, and MMP9 were the core targets of SHD in preventing and treating cisplatin-induced AKI. The main compound bound to the core protein was further confirmed by molecular docking.

The predictive power of network pharmacology was further confirmed *in vivo*, suggesting that SHD decoction can prevent and treat cisplatin-induced AKI. It mainly exerts an anti-inflammatory effect mainly by regulating various pro-inflammatory and anti-inflammatory cytokines and exerts an anti-apoptotic effect mainly through the intrinsic and extrinsic apoptosis pathway.

SHD exerts significant anti-apoptosis and anti-inflammatory activities to attenuate cisplatin-induced kidney damage. Our study shows that SHD regulates the MMP9/PI3K/AKT anti-apoptosis pathway. MMPs family genes are abnormally expressed in the most common kidney diseases. In animal models of AKI induced by folic acid and ischemia/reperfusion (I/R), MMP9 activity was significantly downregulated. It could be activated through the soluble stem cell factor (SCF)/c-KIT pathway, and MMP9-mediated shedding of membrane SCF had a renoprotective effect in acute kidney injury. SCF binds to c-Kit, which induces anti-apoptotic signaling through the PI3K-AKT1 kinase (Arnould et al., 2009). MMP9 knockout mice have reduced SCFs, more severe kidney damage, and increased apoptosis than control mice, and renal function is not easy to recover (Bengatta et al., 2009). Interestingly, PI3K is dependently related to MMPs (Liang et al., 2021). PI3K activation can activate the downstream protein AKT, and the activated AKT can regulate a variety of downstream arnouapoptosis-related genes, among which Bad (Bcl-2-related cell death agonist) can positively regulate mitochondrial apoptosis. After AKT phosphorylates this protein, Bad activity is inhibited, and anti-apoptosis factor Bcl-2 activity is increased (Fulda, 2014). The experimental results showed that cisplatin-induced AKI inhibited the activity of MMP9 to inhibit the PI3K/AKT pathway, thereby promoting apoptosis, and SHD successfully reversed this phenomenon. However, in I/R-induced AKI mice, MMP9 deletion preserves tissue vascular endothelial growth factor levels and stabilizes microvascular density to protect against renal injury. (Lee et al., 2011). This study indicates that MMP9 is special in the pathology of AKI, which is worth exploring in further study.

There are two pathways of apoptosis in mammalian cells: the “extrinsic pathway” mediated by cell surface death receptors, and the other, the “intrinsic pathway” dominated by mitochondria. Caspase-3 is considered to be a common effector of exogenous and endogenous apoptosis. Studies have shown that inhibiting the activity of caspase-3 can inhibit cisplatin-induced apoptosis, which is in line with the results of network pharmacology (Em et al., 2015). Our experimental results indicated that the intrinsic and extrinsic apoptosis pathways are involved in cisplatin-induced AKI and play an essential role in its development. Because SHD exerts its anti-apoptosis through multi-targets and multi-ingredients. Mitochondria are the central organelle where AKT exerts its anti-apoptosis effect. To induce apoptosis, the mitochondrial outer membrane must become permeable so protein can escape. Bcl-2 is closely related to the release of mitochondrial intermembrane proteins. AKT participates in the regulation of different stages of mitochondria-mediated apoptosis by regulating Bcl-2. Cisplatin inhibits the PI3K/AKT pathway while activating the translocation of caspase-3, caspase-9, and Bax to mitochondria, resulting in cell expansion c, apoptosis-inducing factor (AIF), and endonuclease G release. Inhibition of caspase-3 and caspase-9 inhibits cisplatin-induced cell death. In contrast, cisplatin-treated Bax-deficient mice had reduced cytochrome c release and reduced apoptosis (Volarevic et al., 2019). We observed that SHD significantly attenuated the pathological damage of renal tubular cell apoptosis and that SHD significantly regulated the critical targets of the apoptosis pathway, Bcl2, caspase-3, caspase-9, and Bax. Furthermore, in cisplatin-AKI, TNF-α induced extrinsic apoptosis *via* caspase-8, which was also alleviated after SHD treatment.

Inflammation is an essential feature of the pathogenesis of AKI, in which the NF-κB signaling pathway is triggered by renal tubular epithelial cell injury by controlling the expression of pro-inflammatory factors, including IL-1β and IL-6. On the other hand, anti-inflammatory factors also play an important role in protecting renal function and inhibiting AKI. Anti-inflammatory factors, like IL10, can inhibit inflammation and tissue injury by suppressing migration, accumulation, and activation of monocytes and neutrophils. IL-10 can also significantly inhibit the cytotoxicity, inflammation, and apoptosis of renal injury (Zeinali et al., 2022). Other anti-inflammatory factors, such as IL13, activates M2 macrophages, which can promote the tissue repair and remodeling of acute nephritis by secreting various anti-inflammatory mediators, such as IL-4 and IL-10 (Mao et al., 2020). Studies have shown that cisplatin induces a cascade of inflammatory reactions with increased production of proinflammatory cytokines, particularly TNF-α, which has a central role in mediating cisplatin-induced inflammatory renal injury (EM et al., 2015). Inhibiting the expression of TNF-α can significantly attenuate renal injury induced by cisplatin. Meanwhile, TNF-α also plays a crucial role in renal tubular inflammation and is the upstream initiation stage of the inflammatory cascade.

Our previous findings (He et al., 2022) showed that inflammation and apoptosis are closely related to NF-kB signaling. Its’ activation is dependent on the phosphorylation of p65 and inhibitory IκB proteins. Data from our study suggest that SHD regulates the expression of NF-κB-related genes that are up or down-regulated in response to cisplatin induction. SHD can inhibit the secretion and emancipation of inflammatory factors such as TNF-α, IL-6, and IL-1β to protect renal tubules from damage, which is just opposite to the nephrotoxicity of cisplatin. Therefore, we confirmed the role of the NF-κB signaling pathway.

This study has certain limitations. First, public databases may not include some compounds and target genes due to the generality of network pharmacology research. Second, the clinical feasibility of SHD for cisplatin-induced AKI requires validation. Third, we found that MMP9 plays a special role in AKI pathology, and further *in vitro* and *in vivo* experiments are needed to determine this.

## Conclusion

In conclusion, this study provided evidence that SHD plays a critical role in anti-inflammation and anti-apoptosis *via* inhibiting the NF-κB signaling pathway and activating PI3K/AKT anti-apoptosis pathway, indicating that SHD is a candidate herbal drug for further investigation in treating cisplatin-induced AKI. This is the first study to analyze the potential main compounds, targets comprehensively, and pathways of SHD in the prevention and treatment of AKI through network pharmacology, molecular docking, and experimental validation to provide supplementary therapy and additional support for AKI. Drug mechanism provides research ideas and foundations.

## Data Availability

The datasets presented in this study can be found in online repositories. The names of the repository/repositories and accession number(s) can be found in the article/Supplementary Material.
